# Perception of dog health and attitudes towards BOAS grading among Danish owners of French bulldog

**DOI:** 10.3389/fvets.2025.1605505

**Published:** 2025-09-15

**Authors:** Helle Friis Proschowsky, Svenja Springer, Sisse Lotze, Frederikke Rafn, Zenia Sandahl, Camilla Sichlau Bruun, Merete Fredholm, Thomas Bøker Lund, Jakob Lundgren Willesen, Peter Sandøe

**Affiliations:** ^1^Department of Veterinary and Animal Sciences, University of Copenhagen, Frederiksberg, Denmark; ^2^Messerli Research Institute, Department of Interdisciplinary Life Sciences, University of Veterinary Medicine, Vienna, Austria; ^3^Department of Food and Resource Economics, University of Copenhagen, Frederiksberg, Denmark; ^4^Department of Veterinary Clinical Sciences, University of Copenhagen, Frederiksberg, Denmark

**Keywords:** BOAS, French bulldog, brachycephaly, healthy breeding, extreme conformation

## Abstract

**Introduction:**

The French bulldog is among the most popular dog breeds in many countries, but the breed is also commonly affected by a range of health problems. A key issue for the breed is Brachycephalic Obstructive Airway Syndrome (BOAS), and a proposed way to improve this situation in the future is selection based on a functional test known as BOAS grading. However, making effective changes in breeding requires a demand for less sick dogs and discrepancies between objective health parameters and owner-perceived health status are well-known among owners of brachycephalic dogs.

**Methods:**

In this survey-based study, we recorded the health problems reported by French bulldog owners as well as their overall perception of health at both breed and individual dog level. We also examined how the presence of health problems influenced the owners’ plan to reacquire or recommend the breed. Finally, we investigated the French bulldog owners’ knowledge of and attitudes towards BOAS grading as a proposed means to enhance respiratory health in the breed.

**Results:**

The questionnaire resulted in 452 completed responses and the most commonly reported health problems were allergies (36.7%), breathing problems (29.6%), and back problems (19.0%). In total, 56% of the owners stated that French bulldogs are less healthy than other breeds, while 64% considered their own French bulldog to be healthier than other individuals of the same breed. Owners reporting that their dog had breathing problems were more likely to also report that their dog snored when awake, had heat intolerance, and was wheezing. The higher the level of experienced health problems, the less likely the owners were to reacquire the breed or recommend it to others. Two thirds of the owners had heard about BOAS grading and among the 300 respondents who wished to reacquire a French bulldog, 79% expressed that they likely (36.7%) or very likely (42.3%) would prefer a puppy from BOAS-graded parents.

**Discussion:**

In line with previous studies, we found a high disease load among French bulldogs as well as a tendency to rate one’s own dog healthier than the average. The high level of reported BOAS symptoms indicates that owners can recognize the symptoms, yet relatively few respondents consulted a veterinarian about breathing problems – indicating some degree of normalization. However, the generally positive attitude toward BOAS grading may forecast a change of priorities among future owners of the breed.

## 1 Introduction

The French bulldog is one of the most popular dog breeds in many countries around the world (1–3). The brachycephalic appearance is attractive to many humans, and selective breeding for a short, rounded head with large, protruding eyes is not restricted to dogs but is also found in other companion animals like cats and rabbits (4, 5). According to a British study, appearance was the factor that most influenced owners of brachycephalic dogs to acquire their breed, followed by suitable breed size, and a perception that the breed is good with children and generally makes a good companion dog (6). The perceived health of the breed was seen to be less influential in decision making among brachycephalic dog owners compared to owners of non-brachycephalic dog breeds (6, 7).

The characteristic cranial conformation and short snout of the brachycephalic dog breeds leave reduced space for internal soft tissue, such as the soft palate and the tongue, and this can lead to obstruction of the airways (8, 9). This results in a predisposition to breathing difficulties and exercise intolerance in brachycephalic dogs. When these problems get to a very severe level of respiratory compromise, they are collectively known as Brachycephalic Obstructive Airway Syndrome (BOAS) (9). The prevalence of BOAS in the three classic brachycephalic breeds (English bulldog, French bulldog and pug) is high. Approximately 50% of dogs from the three breeds were BOAS-affected when the respiratory function was measures by whole-body barometric plethysmography (WBBP) in a UK study (10). A study of upper respiratory tract disorders based on electronic patient records from veterinary practices in England within the VetCompass Program found prevalences of 19.5, 20.0 and 26.5% for English bulldog, French bulldog, and pug, respectively (8). In addition, French bulldogs were highly predisposed to BOAS with an odds ratio of 30.9 in a study based on random sampling of French bulldogs and non-French bulldogs under primary veterinary care in England (11).

Several studies have shown that many owners fail to recognize or react to clinical signs of BOAS because features like snoring and wheezing are considered “charming” and a normal breed characteristic rather than symptoms of disease (7, 12). A similar effect has been found in studies of obesity, where both parents and dog owners tend to underestimate weight or body condition score (BCS) when asked to evaluate their own children or dogs (13, 14). One-third of the dog owners in a Swedish study underestimated the body composition of their own obese dogs but responded well to a standardized education program and were subsequently able to assess BCS comparable to veterinary health care personnel (14). Regarding BOAS, a study from the UK found that although it was possible to improve public understanding and awareness of BOAS through educational interventions, normalization of its clinical signs was still prevalent (15).

In addition to BOAS, French bulldogs are also predisposed to a range of other diseases. These include dermatological problems like allergies and skin fold dermatitis (16, 17), spinal diseases including intervertebral disk disease, congenital vertebral malformations, and tail malformation (18, 19), eye diseases including corneal ulcers, prolapse of the nictitans gland, and entropion (17, 20, 21), and problems related to reproduction, especially an increased risk of dystocia (22). High veterinary costs and reduced life span are some of the consequences of the high disease burden of French bulldogs (23, 24), yet many owners state that they would acquire the breed again in future (7).

Public awareness and concern about the health and welfare of purebred dogs has increased in recent decades, particularly fueled by the BBC1 television documentary ‘Pedigree Dogs Exposed’, which aired in August 2008 (25). In 2024, a Swedish study found a high level of awareness among dog owners, breeders, show judges, and veterinarians of conformation-related health issues in brachycephalic dogs (26). Most participants in the survey agreed that the health of brachycephalic breeds is compromised and that measures should be taken to improve the situation.

The organized dog world has been criticized for not doing enough to ensure healthy breeding and the majority of show judges in the Swedish study agreed either fully or partly that it is essential to follow the breed standard, even if it can be associated with health issues related to their physical appearance, while all other stakeholders mostly disagreed (26). The debate about extreme conformation in dogs has emerged in many countries and new legislation concerning dog breeding has been enforced in countries like the Netherlands (27), Germany (28, 29), Austria (30), and Denmark (31). The Norwegian Supreme Court recently concluded that further breeding of cavalier King Charles spaniels with the current genetic pool is in violation of the Animal Welfare Act, and that English bulldogs must be bred under a breeding program aimed at reducing the occurrence of diseases such as BOAS (32).

Various screening models have been developed with the hope of moving the brachycephalic breeds in a healthier direction by selective breeding. The test most widely adopted internationally is the Respiratory Function Grading (RFG) scheme developed by Jane Ladlow and coworkers at the University of Cambridge (33). This test consists of a physical examination that includes grading nostril stenosis, auscultation of respiratory sounds, a 3-min exercise test, and repeated auscultation. After the test, the dog is assigned one of four grades: 0 (BOAS free), 1 (mild BOAS), 2 (moderate BOAS), or 3 (severe BOAS) (34). BOAS grading can only be performed by veterinarians who have attended a qualifying course. The RFG scheme is currently implemented in Australia, Austria, Belgium, the Czech Republic, Denmark, Germany, Hungary, Iceland, Ireland, Mexico, the Netherlands, New Zealand, Norway, Portugal, Romania, Sweden, Switzerland, the UK, and the US (35, 36). The International organization for dogs, Fédération Cynologique Internationale (FCI), also supports the RFG scheme (37). The Danish Kennel Club (DKC) implemented a mandatory breeding program based on BOAS grading from August 1, 2023. After this date, English bulldog, French bulldog, and pug must have a BOAS grade of 0 or 1 prior to mating for their offspring to obtain a DKC pedigree (38). After July 1, 2025, when new Danish legislation came into force, all dogs belonging to the three breeds used for breeding must have a BOAS grade of 0 or 1 regardless of pedigree (31).

The Finnish Kennel Club (FKC) uses a BOAS test that is slightly modified from the RFG scheme and includes auscultation for respiratory sounds before and after a brisk, time-limited 1,000 m walk, evaluation of nostril stenosis, registration of body temperature, and measurement of the craniofacial ratio (9) to evaluate muzzle length. As part of a validation of the Finnish BOAS scheme, heritability estimates for the severity of BOAS were calculated for parent–offspring pairs of English bulldogs, French bulldogs, and pugs with at least one BOAS-graded parent (h^2^ = 0.39) or both parents BOAS graded (h^2^ = 0.48) (39). These heritability estimates indicate that the Finnish system for BOAS grading has the potential to be used as a selection tool to obtain less-affected offspring.

Supplementary tools like open breed registries or cross breeding projects for brachycephalic dog breeds have been introduced in some countries. When a breed has an open breed registry, it is possible to introduce new individuals without a pedigree to the organized pedigree dog registry. In Denmark, the English bulldog, French bulldog and pug breed registries were opened by May 1, 2024. To enter the Danish Kennel Club registry, a dog must have a BOAS grade of 0 or 1 and its phenotype must be evaluated and approved by an official show judge (40). One of the motivations behind open breed registries is to increase the gene pool of the pedigree population. ID marking and registration in the Danish Dog Registry (DDR) is mandatory in Denmark (41), and data from the DDR and registration figures provided by the DKC show that the immense popularity of the French bulldog has led to an increasing proportion of the Danish population of French bulldogs being bred outside the kennel club. In 2024, a total of 1,531 French bulldogs were registered in the Danish Dog Registry, but only 165 (11%) had a pedigree from the DKC (42, 43).

In Finland, the FKC has introduced several cross breeding projects in recent years, one of which involves the French bulldog, with a statement on the FKC website that *“The aim of the cross breeding project is to improve the breed’s health status and to decrease the exaggerated traits in the appearance of the breed”* (44). The suggested breeds for cross breeding with the French bulldog are: American Staffordshire terrier, Jack Russell terrier, mittel spitz, Papillon/Phalene, Danish-Swedish farmdog, and Staffordshire bull terrier (44). The offspring of deliberate cross breeding projects are normally registered in a separate register in the kennel club and introduced to the purebred breeding population according to defined criteria, which can include health screening results or phenotypic evaluation.

The focus of the present study is on Danish dog owners’ perceptions of breed-related health problems in French bulldogs and their attitudes toward BOAS grading and the specific aims were to: (1) report the prevalence of specific health problems among the dogs of respondents; (2) assess how problematic to the breed the owners find each health issue; (3) determine the extent to which owners have consulted a veterinarian about the health issues; (4) examine how respondents perceive the health of French bulldog compared to other breeds, as well as the health of their own dog compared to other French bulldogs, and how this is influenced by owner characteristics and the disease load of the respondent’s own dog; (5) identify the prevalence of eight specific clinical signs associated with BOAS and their correlation with the owners’ perception of their dog having breathing problems; and investigate how owner characteristics, the general perceived health status of the breed, the perceived health status of their own dog, and the owner’s attitude toward BOAS grading influence the (6) likelihood of owners’ plan of having their current dog BOAS-graded in the future and preferring a puppy from BOAS-graded parents; and (7) the likelihood of acquiring another French bulldog or recommending French bulldogs to others. The part of the present study that relates to perceptions of health is very close to a previous UK study that used almost the same approach and set of questions although the UK study was bigger and covered more breeds (45, 46).

## 2 Materials and methods

The study was part of a larger study that also included breeders. Unfortunately, not enough answers were received from breeders to allow for a proper analysis. Therefore, the questions aimed at breeders were excluded from the present study.

### 2.1 Participants

The inclusion criteria for participants of this study were being a Danish dog owner at least 18 years old and owning at least one living French bulldog. The link to the online questionnaire was shared in eight Facebook groups specifically relating to French bulldog and on the Facebook page of the DKC. The total number of members in the breed-specific Facebook groups was 38,200 and the Facebook page of the DKC had 42,000 members. However, it is likely that many individuals belonged to several groups. In addition, the link could have been shared in other Facebook groups, making it challenging to estimate its reach accurately.

Participants with more than one French bulldog were asked to complete the questionnaire based on the most recently acquired dog. This was to avoid bias caused by choosing either the dog that the owner thought was healthiest, or conversely the dog that the owner believed had the greatest number of problems.

### 2.2 Questionnaire development

#### 2.2.1 Qualitative interviews

Qualitative, semi-structured interviews with owners and breeders of pedigree and non-pedigree French bulldogs were carried out in September 2023 prior to designing the questionnaire. There were nine interviewees in total, representing five breeders and four owners of French bulldog, but only the latter group was relevant to this study. The interviews were used to identify the owner’s knowledge of the health problems in the breed and clinical signs of BOAS, and to assess awareness of and opinions about various initiatives to promote healthy breeding in brachycephalic dogs. An English translation of the interview guide is provided as Supplementary material 1.

#### 2.2.2 Development of the questionnaire based on interview data

The final questionnaire was designed in SurveyXact (47) and comprised 59 questions, of which 27 are included in this study, covering: (1) sociodemographic information about the owners and characteristics of their dogs; (2) opinions on reacquiring the breed or recommending it to others; (3) personal experience with and general perception of specific health problems; (4) perceptions of the health of the breed in general and of the individual dogs; (5) questions related to specific clinical signs of impaired breathing; (6) knowledge and opinions about BOAS grading. The remaining part of the questionnaire was aimed at breeders and will as already mentioned not be reported. An English translation of the relevant part of the questionnaire is provided as Supplementary material 2.

A pilot test of the questionnaire was sent out to 11 dog owners before the distribution. The final questionnaire was available from Oct 05 to Oct 25, 2023.

### 2.3 Data analysis

We used the statistical software IBM® SPSS Statistics version 29.0 (IBM® SPSS® Statistics, Chicago, IL, United States) for all analyses. Univariable descriptive and multivariable statistics (binary and ordinal regression analyses) were presented in tables or text. For continuous variables, median values were reported along with interquartile ranges to provide a more robust representation of the data, especially in light of potential skewness or the presence of outliers. For statistical tests, *p*-values below 0.05 were considered significant. To address the increased risk of Type I errors (false positives) arising from multiple hypothesis tests in our regression models, the Bonferroni correction was applied to adjust the significance level. This adjustment helps control for the cumulative risk associated with multiple comparisons, ensuring that the findings are robust and reducing the likelihood of mistakenly identifying a predictor as significant when it is not. The Bonferroni correction was employed for multiple comparisons. Detailed explanations can be found in the notes accompanying the tables where the Bonferroni corrections were applied.

For the analysis of research aim 4, the dependent variables were the (1) owners’ estimation of the health status of French bulldog compared to other breeds and (2) owners’ estimation of the health status of their own dog compared to other French bulldogs. In both questions the owners were asked to place their own dog on a scale from 1 to 5, where 1 = much less healthy, and 5 = much healthier. Two ordinal regression analyses were conducted with the following explanatory variables included as continuous variables: age of the owner; total score of health problems from question S_82 – S_89 [range from 0 to a max. of eight perceived health problems]; cost of the dog. The gender of the owner [1 = male, 2 = female] and whether it was the owner’s first French bulldog [1 = yes, 2 = no] were included as categorical variables. Responses of “I do not know” were excluded from the analysis.

For the analysis of research aim 5, a binary logistic regression analysis was conducted to examine the impact of eight clinical signs of BOAS (question S_123 – S_130 in the questionnaire, yes/no) on whether dog owners indicated that their dog is suffering from breathing problems [question S_86 in the questionnaire (yes/no)]. All clinical signs were included in the model as independent variables to account for the presence of multiple signs in the study population. The dependent variable in this analysis was the owners stating whether their dog had suffered from breathing problems, coded as 0 for no breathing problems and 1 for the presence of breathing problems.

Two ordinal regression analyses were conducted for the analysis of research aim 6. The dependent variables were (1) having the current dog BOAS graded in the future, and (2) preferring a puppy from BOAS graded parents when acquiring another French bulldog [1 = very unlikely to 5 = very likely]. The following factors were included as continuous, explanatory variables: owners’ age, total score of perceived BOAS-related health problems, owners’ estimation of the health status of French bulldog compared to other breeds, and owners’ estimation of the health status of their own dog compared to other French bulldogs [1 = much less healthy to 5 = much healthier]. Additionally, two statements reflecting owner attitudes toward BOAS grading were included: (1) BOAS grading is a good initiative and (2) BOAS grading should be mandatory for all French bulldogs used for breeding, regardless of pedigree [1 = completely disagree to 5 = completely agree].

For the analysis of research aim 7, the dependent variables were (1) acquiring another French bulldog and (2) recommending a French bulldog to others, two ordinal regression analyses were conducted [1 = very unlikely to 5 = very likely]. The following factors were included as continuous, explanatory variables: owners’ age, total score of perceived BOAS-related health problems, owners’ estimation of the health status of French bulldog compared to other breeds, and owners’ estimation of the health status of their own dog compared to other French bulldogs [1 = much less healthy to 5 = much healthier]. Additionally, two statements reflecting owner attitudes toward BOAS grading were included: (1) BOAS grading is a good initiative and (2) BOAS grading should be mandatory for all French bulldogs used for breeding, regardless of pedigree [1 = completely disagree to 5 = completely agree].

## 3 Results

A total of 653 responses were received from Danish owners of French bulldogs, of which 452 were completed questionnaires.

The majority of the responding dog owners were female (*N* = 411, 90.9%) and more than half of the responses came from owners who were either between 35 and 44 years old (*N* = 114, 25.2%) or between 45 and 54 years old (*N* = 139, 30.8%).

The distribution of male and female dogs was 255 (56.4%) male and 197 (43.6%) female dogs. With 49.6% (*N* = 224), half of the dogs were aged between 1 and 3 years, while about one-third fell within the 4 to 9-year age range (*N* = 153, 33.8%). Additionally, 10.6% (*N* = 48) of the dogs were younger than 1 year, and 6.0% (*N* = 27) were aged 10 years or older, with the oldest being 14 years. Approximately two thirds of the dogs were intact (*N* = 288, 63.7%), while 164 (36.3%) were neutered. A total of 65.0% (*N* = 294) of the 452 dog owners stated that the dog in question was their first French bulldog.

### 3.1 Owner-reported prevalence of eight health problems in French bulldogs

In total, 157 owners (34.7%) reported that they perceive none of the provided health problems in their dog. Among those who reported health problems, the most common owner-reported health problem was allergies, affecting more than one third of dogs in the study (*N* = 166, 36.7%). Breathing problems (*N* = 134, 29.6%) and diseases of the spinal cord (*N* = 86, 19.0%) were the second and third most common issues, respectively (Table 1). Among the 452 dogs studied, the median number of reported health problems was 1, with an interquartile range of 0 to 2. Among the 295 dog owners (65.3%) who reported perceiving health problems in their dogs, the median number of perceived health issues was 2, accompanied by an interquartile range of 1 to 3.

**Table 1:** Prevalence of owner-reported health issues among 452 dogs, sorted by decreasing prevalence.

Health problem	Number of dogs (percentage)
Allergies	166 (36.7%)
Breathing problems	134 (29.6%)
Spinal diseases	86 (19.0%)
Eye problems	80 (17.7%)
Skin fold infections	73 (16.2%)
Hip problems	29 (6.4%)
Knee problems	25 (5.5%)
Reproductive problems	18 (4.0%)

### 3.2 Perception of health problems in the breed

The respondents were asked to state whether the eight health problems from Table 1 could be considered as generally problematic for the breed regardless of the health status of the respondent’s own dog. More than half of the dog owners considered breathing problems (*N* = 255, 56.4%) and allergies (*N* = 253, 56.0%) as generally problematic for the breed to a high or a very high degree. Knee problems were considered the least problematic health issues among the respondents (*N* = 68, 15.0%). Table 2 shows the extent to which the 452 respondents perceived the specific diseases as problematic for the breed in general by decreasing percentage of “to a high degree” and” to a very high degree” combined.

**Table 2:** Perception of the degree to which eight health issues are considered problematic for the breed in general according to 452 respondents, sorted by decreasing percentage of “to a high degree” and “to a very high degree” responses combined.

Health problem	Not at all severe	To a lesser extent	To some extent	To a high degree and to a very high degree (combined)
Breathing problems	25 (5.5%)	50 (11.1%)	122 (27.0%)	255 (56.4%)
Allergies	39 (8.6%)	48 (10.6%)	112 (24.8%)	253 (56.0%)
Spinal diseases	58 (12.8%)	63 (14.0%)	162 (35.8%)	169 (37.4%)
Reproductive problems	87 (19.2%)	83 (18.4%)	170 (38.6%)	112 (24.8%)
Skin fold infections	64 (14.2%)	137 (30.3%)	151 (33.4%)	100 (22.1%)
Hip problems	72 (15.9%)	120 (26.5%)	168 (37.2%)	92 (20.4%)
Eye problems	88 (19.5%)	148 (32.7%)	147 (32.5%)	69 (15.3%)
Knee problems	77 (17.0%)	145 (32.1%)	162 (35.8%)	68 (15.1%)

### 3.3 Veterinary consultations

When the respondents reported the presence of a specific health issue in their dog, they were also asked to state whether or not they had consulted a veterinarian about it. The results are summarized in Table 3, which shows the percentage of dog owners who had or had not sought veterinary consultancy in each disease category, listed as the highest to the lowest percentage of “Yes” responses.

**Table 3:** Veterinary consultation among the 452 respondents for each of the eight health issues, listed from the highest to the lowest percentage of “Yes” responses.

Health problem	N	“Yes” to consulting a veterinarian	“No” to consulting a veterinarian
Spinal diseases	86	81 (94.2%)	5 (5.8%)
Allergies	166	155 (93.4%)	11 (6.6%)
Eye problems	80	71 (88.8%)	9 (11.3%)
Knee problems	25	22 (88.0%)	3 (12.0%)
Reproductive problems	18	15 (83.3%)	3 (16.7%)
Hip problems	29	24 (82.8%)	5 (17.2%)
Skin fold infections	73	52 (71.2%)	21 (28.8%)
Breathing problems	134	81 (60.4%)	53 (39.6%)

Although most respondents perceived breathing problems as problematic to a high or very high degree, it was also the condition for which the fewest owners sought veterinary care. Only 81 (60.4%) of the 134 owners who reported that their dog suffered from breathing problems indicated that they had consulted a veterinarian. For comparison, spinal diseases and allergies resulted in more than 90% (81 out of 86 and 155 out of 166 respectively) of the owners visiting a veterinarian.

### 3.4 Owners’ perception of the health of the French bulldog breed in general and their own dog

More than half of the owners stated that French bulldog is generally less healthy (*N* = 211, 46.7%) or much less healthy (*N* = 41, 9.1%) than other breeds, while 63.9% (*N* = 289) considered their own dog to be either healthier (*N* = 153, 33.8%) or much healthier (*N* = 136, 30.1%) than other French bulldogs (Table 4).

**Table 4:** The perception of breed health in general and the health of the owners’ own dogs, according to 452 French bulldog owners.

Perception of health	Much less healthy	Less Healthy	Average	Healthier	Much healthier	Do not know
French bulldog compared to other breeds	41 (9.1%)	211 (46.7%)	173 (38.3%)	12 (2.7%)	3 (0.7%)	12 (2.7%)
The respondent’s own dog compared to other French bulldogs	8 (1.8%)	29 (6.4%)	122 (27.0%)	153 (33.8%)	136 (30.1%)	4 (0.9%)

Owners who observed fewer health problems in their dogs were more likely to state that French bulldog is healthier than other breeds (*p* < 0.001) in general and that their own dog is healthier than other French bulldogs (*p* < 0.001; Supplementary material 3). None of the other explanatory variables (age of the owner, cost of the dog, gender of the owner, and whether it was the owner’s first French bulldog) were significant.

### 3.5 Prevalence of eight clinical signs of BOAS and their association with the owner stating that their dog has suffered from breathing problems

In question S_123 to S_130, the respondents were asked to indicate whether they had experienced any of the eight specific behaviors or clinical signs of BOAS listed in Table 5 in their dogs. Snoring when asleep was reported for nearly three in four dogs (*N* = 328, 72.6%) and more than half of the dogs grunted (*N* = 282, 62.4%) or regurgitated/vomited (*N* = 233, 51.5%). Three of these behaviors or signs were significantly associated with owners answering “yes” to question S_86 “has your dog suffered from breathing problems” (Table 5). Specifically, owners reporting that their dog had breathing problems were more likely to also report that their dog snored when awake (*p* < 0.001), had heat intolerance (*p* < 0.001), and was wheezing (*p* = 0.005).

**Table 5:** Prevalence of behaviors and/or clinical signs of respiratory impairment, odds ratios with 95% confidence intervals, and *p*-values of their association with the owners stating that their dog has suffered from breathing problems.

Clinical sign	Prevalence	OR (95% CI)	*p*-value^*^
Snoring when asleep	328 (72.6%)	1.49 [0.74–2.97]	0.264
Grunting	282 (62.4%)	2.25 [1.22–4.15]	0.010
Regurgitation/vomiting	233 (51.5%)	1.45 [0.85–2.48]	0.173
Heat intolerance	217 (48.0%)	3.78 [2.22–6.43]	**<0.001**
Exercise intolerance	73 (16.2%)	1.39 [0.71–2.74]	0.340
Snoring when awake	71 (15.7%)	5.71 [2.89–11.26]	**<0.001**
Wheezing	55 (12.2%)	3.13 [1.42–6.89]	**0.005**
Sleep problems	12 (2.7%)	14.25 [1.32–154.37]	0.029

*Bonferroni correction was applied for significant results; as there are eight tests being made, one for each clinical sign, alpha was divided by 8 (*N* = 8): 0.05/8 = 0.006, i.e., each test is tested against a level of 0.006. Significant *p*-values are highlighted in bold.

### 3.6 Knowledge and perception of BOAS grading

Respondents fell into three almost equal groups with respect to knowledge about the implementation of mandatory BOAS grading in pedigree dogs: 32.3% (*N* = 146) indicated that they were well informed; 33.4% (*N* = 151) had heard about it but did not know much about it in detail, and 34.3% (*N* = 155) had never heard of BOAS grading.

The perception of BOAS grading was generally positive. A total of 85.0% (*N* = 384) agreed that BOAS grading was a good initiative and 81.4% (*N* = 368) agreed that it has the capacity to improve the health of the breed. The vast majority of respondents (*N* = 378, 83.6%) agreed or completely agreed that BOAS grading should be mandatory for all French bulldogs and not only dogs with a pedigree from the DKC. In addition, 60.7% (*N* = 274) agreed or completely agreed that BOAS grading should be regulated via national legislation. However, it is worth noting that 60 respondents (13.3%) answered “Do not know” to this question (Table 6).

**Table 6:** Level of agreement with various statements about BOAS grading among the 452 respondents.

Statement	Completely agree	Agree	Neither agree nor disagree	Disagree	Completely disagree	Do not know
BOAS grading is a good initiative	318 (70.4%)	66 (14.6%)	34 (7.5%)	8 (1.8%)	9 (2.0%)	17 (3.8%)
BOAS grading has the capacity to improve the health of the breed	270 (59.7%)	98 (21.7%)	34 (7.5%)	12 (2.7%)	7 (1.5%)	31 (6.9%)
BOAS grading should be mandatory for all French bulldogs used for breeding regardless of pedigree	334 (73.9%)	44 (9.7%)	36 (8.0%)	3 (0.7%)	12 (2.7%)	23 (5.1%)
BOAS grading should be regulated by legislation	201 (44.5%)	73 (16.2%)	75 (16.6%)	8 (1.8%)	35 (7.7%)	60 (13.3%)

The majority of the respondents stated that their own dog had not been BOAS graded (*N* = 377, 83.4%). The likelihood that these 377 dogs would be BOAS graded in the future is presented in Table 7 alongside the likelihood of prioritizing a puppy from BOAS-graded parents according to the 300 respondents who stated that they would likely or very likely acquire another French bulldog. Less than one third (*N* = 108, 28.6%) indicated that they were likely or very likely to have their current dog BOAS graded, while nearly half of the respondents (*N* = 178, 47.4%) stated that they were unlikely or very unlikely to do so. In terms of acquiring a new puppy, the situation looks quite different, where 79% (*N* = 237) of respondents stated that they are likely or very likely to prefer a puppy from BOAS-graded parents when acquiring their next French bulldog.

**Table 7:** Likelihood of BOAS grading their current dog according to 377 owners of non-BOAS-graded dogs and the likelihood of prioritizing a puppy from BOAS-graded parents according to 300 respondents who stated that they wished to acquire another French bulldog.

Likelihood	Likelihood of having the current dog BOAS graded in the future (*N* = 377)	Likelihood of preferring a puppy from BOAS-graded parents when acquiring another French bulldog (*N* = 300)
Very likely	46 (12.2%)	127 (42.3%)
Likely	62 (16.4%)	110 (36.7%)
Neither likely nor unlikely	60 (15.9%)	47 (15.7%)
Unlikely	68 (18.0%)	9 (3.0%)
Very unlikely	111 (29.4%)	7 (2.3%)
Do not know	30 (8.0%)	0

The likelihood of having the current dog BOAS graded in the future increased with a higher level of agreement that BOAS grading should be mandatory for all French bulldogs used for breeding, regardless of pedigree (*p* < 0.001; Supplementary material 4). Furthermore, with increasing age, owners became less likely to have their current dog BOAS graded in the future (*p* < 0.001).

The likelihood of preferring a puppy from BOAS-graded parents when seeking a new French bulldog increased with a higher level of agreement that BOAS grading is a good initiative (*p* < 0.001), and that BOAS grading should be mandatory for all French bulldogs used for breeding regardless of pedigree (*p* < 0.001; Supplementary material 4).

### 3.7 Reacquisition and recommendation of French bulldog

Respondents who indicated plans to acquire a new dog in future (*N* = 370) were asked about their attitudes toward acquiring another French bulldog. All 452 respondents were also asked how likely they were to recommend the breed to others. In total, 81.1% (*N* = 300) of the French bulldog owners who planned to acquire a new dog indicated that it was very likely (*N* = 210, 56.8%) or likely (*N* = 90, 25%) that they would purchase a dog of the same breed again (Table 8). Fewer—but still a majority—would very likely (*N* = 199, 44.0%) or likely (*N* = 105, 23.2%) recommend the breed to others.

**Table 8:** The likelihood of acquiring another French bulldog among the 370 respondents who indicated that they planned to acquire a new dog in future, and the likelihood of recommending French bulldog to others among all 452 respondents.

Reacquiring and recommending	Very likely	Likely	Neither likely nor unlikely	Unlikely	Very unlikely
Likelihood of acquiring another French bulldog (*N* = 370)	210 (56.8%)	90 (25.0%)	24 (6.5%)	35 (9.5%)	11 (3.0%)
Likelihood of recommending French bulldog to others (*N* = 452)	199 (44.0%)	105 (23.2%)	82 (18.1%)	54 (11.9%)	12 (2.7%)

When it comes to factors affecting the likelihood of reacquiring a French bulldog, owners who believed that their dog was healthier than other breeds (*p* < 0.001) were more likely to reacquire a French bulldog.

In terms of recommending a French bulldog to others, owners who observed fewer health problems in their dog (*p* = 0.006), and who believed that French bulldog was healthier than other breeds (*p* < 0.001) were more likely to recommend a French bulldog to others (Supplementary material 5).

## 4 Discussion

This paper presents the results of a questionnaire study among Danish owners of French bulldogs focusing on their perception of the current health status of the breed in general and their own dog specifically, and how this related to their opinion of BOAS grading as a tool to improve respiratory health among French bulldogs in future and their tendency to either reacquire or recommend the breed to others.

The results are in line with other studies (11, 17) and confirm that the French bulldog is a breed with a high disease load. Nearly one third of the dogs in the present study had allergies or breathing problems. Furthermore, over half of the dog owners stated that poor respiratory health is problematic for the breed. More than half of the owners also perceived the French bulldog to be less healthy than other breeds, while 64% considered their own dog to be healthier than other French bulldogs. The individual dog’s perceived health also played a role, as owners who observed fewer health problems in their own dogs were more likely to state that French bulldog is healthier than other breeds and that their own dog is healthier than other French bulldogs. Despite the significant prevalence of health issues, more than 80% of the respondents expressed a desire to own another French bulldog, and over 60% would recommend the breed to others.

Both the tendency to rate one’s own dog healthier than the average and the urge to acquire another brachycephalic dog have also been described in other studies (7, 12). The previously mentioned questionnaire study performed in the UK across the three “classic” brachycephalic dog breeds (English bulldog, French bulldog and pug) reported similar results regarding the most common diseases, yet 70.9% of the 2,168 respondents still considered their own dog to be in very good health or the best health possible (45). The subsequent study based on the same questionnaire data found that 93% of the 2,168 owners were highly likely to acquire their breed again and 65.5% would recommend the breed to other owners (46). The study analyzed factors affecting what has been termed “dog breed loyalty” (48) and concluded: “Although owners are initially attracted to the distinctive appearance of brachycephalic dogs, perceived breed-related behavioral traits are a core component of why owners perceive their breed positively, alongside the strong emotional bonds they inspire” (46). It is relevant to mention that emotional bonds or pet attachment have been shown to influence the ability to evaluate one’s own animal objectively with respect to diseases and traits like BCS. The extent to which owners have favorably distorted views of their pets has been found to be positively correlated with pet attachment (49).

A significant difference between breeds in the formation of owner-dog bonds was also identified in a representative study among Danish owners of four dog breeds (Cairn terrier, cavalier King Charles spaniel, Chihuahua, and French bulldog). The study compared motivational factors behind acquisition, the prevalence of health problems, quality of the owner-dog bond, as well as the intention to acquire another dog of the same breed (7). The Lexington Attachment to Pets Scale (LAPS) (50) was used to quantify the emotional attachment between owners and dogs and the results revealed that higher levels of health and behavioral problems did not appear to have a negative impact on the quality of the owner-dog relationship. On the contrary, high levels of health and behavioral problems in Chihuahuas and cavalier King Charles spaniels were positively correlated with high levels of owner-dog attachment, suggesting that caregiving behavior may reinforce the formation of strong owner attachments. Despite the high health care burden, many owners still intend to reacquire a type of dog that they now personally know is likely to have serious health issues. However, both the present study and the Danish study from 2017 found that as the number of health problems increased, there was a corresponding decrease in the owners’ intention to acquire another French bulldog (7). Apparently, some owners perceive the long-term commitment and potential costs of managing a higher number of health issues as burdensome, which can deter them from acquiring another dog of the same breed.

It has been suggested that pre-purchase consultations by veterinarians could be seen as an opportunity to influence decisions to acquire or re-acquire brachycephalic dogs, but according to a British study, many veterinarians find it difficult to overcome barriers like a fear of negative consequences of discussing brachycephalic health and welfare, including the risk of compromising their relationship with an existing client (51).

There was a high incidence of clinical signs like snoring when asleep (72.6%) and grunting (62.4%) reported in the present study. In addition, nearly half of the dogs presented with heat intolerance, which is likely to affect the owner’s ability to walk their dogs and engage in other outdoor activities during the summertime. The high level of reported BOAS symptoms indicates that owners are able to recognize these symptoms in their dogs, yet relatively few respondents consulted a veterinarian about breathing problems. One possible explanation is that the only treatment available is surgical correction, and financial considerations may discourage some owners from seeking veterinary advice and potential treatment. However, it may also be because some owners underestimate the seriousness of the problem, partly because it is such a common problem in the breed. The results may reflect a form of acceptance of health issues at the individual level, to which owners may adjust their perception based on the general assessment of the breed’s well-known health issues.

A study from 2019 evaluated the correlations between brachycephalic syndrome and phenotypic characteristics like nostril stenosis and neck girth in a population of 69 French bulldogs from Denmark (52). The authors concluded that there is a long way to go before French bulldogs can be bred to breathe well. However, a lot has happened in the dog-breeding community since 2019, in particular with the development and distribution of standardized BOAS-grading tools like the Respiratory Function Grading (RFG) scheme developed by Jane Ladlow and coworkers at the University of Cambridge (33).

Grading of BOAS using the Cambridge RFG scheme only became available in Denmark a short time before the questionnaire of the present study was distributed to owners of French bulldogs. Despite this, only one third of respondents stated that they had never heard about it. This may be attributed to widespread attention from the media when it comes to health problems of purebred dogs, in particular brachycephalic dogs. The present study reveals significant support for BOAS grading with more than 80% of respondents stating that BOAS grading is a good initiative that has the capacity to improve the health of the breed. In addition, 79% of respondents stated that they will prioritize a puppy from BOAS-graded parents when acquiring their next French bulldog.

The effect of using the Cambridge RFG scheme as a tool to improve the health of brachycephalic breeds through selective breeding has not yet been evaluated and has some limitations. Critics argue that the grading system lacks standardization, which can lead to variability in assessments among veterinarians (53). This subjectivity raises concerns about the reliability and consistency of the grading results. Furthermore, the reliance on physical examinations and exercise tests may not adequately capture the full spectrum of respiratory issues faced by brachycephalic dogs, thus presenting challenges in achieving a universally accepted standard (39). In addition, some critics claim that the scheme promotes the breeding of animals that still have some level of respiratory impairment and that the scheme does little to modify well-known conformational risk factors of BOAS like craniofacial ratio, eye width ratio and neck girth ratio (10). As a result, there is ongoing debate within the veterinary community about the effectiveness of the Cambridge RFG scheme and the potential for inconsistencies in its application in breeding decisions. This lack of consensus underscores the necessity for continued research and the development of more objective measures to ensure the welfare of breeds with a high prevalence of BOAS. The heritability estimates, that were calculated from the Finnish version of the BOAS test, seem promising (39). However, to make progress, adequate population size in order to avoid inbreeding (52, 54, 55) in combination with the willingness to prioritize health over other selection criteria are necessary regardless of the method.

### 4.1 Limitations of the study

The present study has some important limitations. Firstly, the data were collected as a convenience sample with Facebook as the primary distribution source. As a result, owners who are not active on Facebook will not have had the chance to fill out the questionnaire. One third of the respondents were between 45 and 54 years old and we therefore missed some younger dog owners maybe because they have moved to other social media and are less active on Facebook. The breed-specific Facebook groups might also attract owners who are more invested in the breed than the average French bulldog owner and who have a greater willingness to improve the health of the breed. This could have inflated an overly positive perception of BOAS grading and underrepresented the share of French bulldogs with severe health problems. In addition, the gender distribution of the respondents was skewed with 90.9% being female which is also a common phenomenon when surveys are distributed via social media like Facebook. Precise information about gender distribution of dog owners is not available from the Danish Dog Registry, but using data from a representative study of pet ownership in Denmark, it was possible to calculate that 56% of the Danish dog owners are female and 44% are male (56). Even though we do not know if this distribution also holds true for French bulldogs, the skewed gender distribution of the present study may be missing a different set of views from male owners.

Secondly, all information about diseases is owner-reported and we do not know if the health issues have been diagnosed by a veterinarian. Some disease categories were broad and covered a range of different diagnoses, while others were narrower. “Allergies” could for instance include both food allergy and atopic dermatitis, while skin fold infections is probably a more uniformly perceived issue. As the owners answered the questionnaire retrospectively and the age of the dogs varied at the time of participation, there may also be an element of recall bias. This may have resulted in inaccuracies in some of these responses. We tried to minimize this by asking owners with more than one French bulldog to answer for the most recently acquired dog, but this will likely have caused a sample skewed toward younger dogs. The convenience sampling means that the reported prevalence cannot be regarded as the ‘true’ prevalence of diseases in the Danish population of French bulldogs.

Thirdly, the description of the BOAS test at S-52 in the questionnaire lacks the information that the result of a BOAS grading cannot be considered static as it can change according to for instance a given dog’s age or body condition score. The questionnaire thereby gives a partial but not full description of the test which may have influenced the responses to the following questions for those who had no prior knowledge of the test.

Problems related to the health and welfare of brachycephalic dog breeds have been subject to intense media coverage in recent years. This opens up the possibility of owners answering the questions in a way that portrays them in a more positive light, in line with what they perceive as socially desirable and acceptable. We have tried to minimize this social desirability bias by formulating the questions in a neutral way.

## 5 Conclusion

To conclude, the present study found that allergies and breathing problems were the two most prevalent diseases among French bulldogs and that more than half of the 452 owners perceived these diseases as problematic for the breed. Even though owners were fully able to identify the clinical signs of breathing difficulties, they seldomly, compared to the other reported diseases, consulted a veterinarian about them. The study reproduces the high level of breed loyalty among owners of French bulldogs seen in other studies as well as a tendency to rate one’s own dog healthier than the average. A change of priorities in pedigree dog breeding is underway in many countries (57), in some cases supported by legislative initiatives and this may be reflected in the generally positive attitude toward BOAS grading as a tool to improve the respiratory health of the French bulldog breed.

## Data Availability

The dataset presented in this study can be found in an online repository at: https://zenodo.org/records/15068495. The interview data will be made available upon reasonable request, conditional on signing a confidentiality requirement.
