# Supplementary material for: Perception of dog health and attitudes towards BOAS grading among Danish owners of French bulldog
Source: Front Vet Sci. 2025 Sep 15;12:1605505. doi: 10.3389/fvets.2025.1605505 (PMC12477691; doi:10.3389/fvets.2025.1605505)
Supplement: Supplementary file 1 [file Data_Sheet_1.pdf]

English translation of the interview guide for owners used for the publication "Perception of health problems in French bulldogs and attitudes towards BOAS-grading among Danish owners".

Introduction	Thank you for participating	We would like to start by thanking you for your participation.
Presentation of the group and purpose	Presentation of the group and distribution of roles	The group consists of Sisse, Zenia and Frederikke. We are all veterinary students at the University of Copenhagen and in the process of writing our master's thesis on French bulldogs.<br><br>Frederikke will ask the questions, and then Sisse and Zenia will take notes and come up with any follow-up questions along the way.
	Purpose of the project	The purpose is to investigate the interest in breeding healthier French bulldogs
Formalities	Schedule	We expect the interview to take about 30-45 minutes.
	Recording of interviews	We must make you aware that the interview will be recorded. Data will be used as a basis for a questionnaire to be included in our thesis and subsequently a scientific article. This recording is for our own use only and to ensure the details of the interview. After data collection and processing, all recordings will be deleted.
	Consent and anonymization	You can withdraw your consent at any time during the interview, and we will stop the interview and delete all information, and it will not be used. After the interview, we make a summary of what we have talked about, and then it is anonymized – that is, we remove your name and all information that can lead back to you. Once the interview has been anonymized, you will no longer be able to withdraw your consent.

	Question	You are welcome to ask questions at any time if there is anything you do not understand or want to elaborate on.
Introduction of the interviewee	The interviewee introduces himself	Would you like to briefly introduce yourself?

**Problem**	**Questions asked**	**What we want to know**
Motivation for buying a French bulldog	1. Tell us a little about your dog and the experiences you have had.<br><br>2. What considerations did you make prior to the purchase?<br><br>3. If you were to get a new dog, is there anything you would do differently?	a. Why did you choose to buy a French Bulldog over another breed?<br>b. What specific characteristics of the breed did you go for?<br>c. How did you familiarize yourself with the breed before buying it, and where did you find this information?<br>d. Where did you buy your dog?<br>e. Does the dog have a pedigree and why did you choose a French Bulldog with/without a pedigree?<br>f. Will you also choose a French bulldog in the future?
Motivation to change the Health of French Bulldogs	1. What health problems in the breed are you familiar with?<br>2. Have you experienced any diseases in your own dog?<br>3. What are your thoughts about these problems?	a. How did you become familiar with these issues?<br>b. What diseases have you experienced in your own dog?<br>c. What have you done to correct these problems in your own dog?<br>d. Have you applied for help with this?<br>e. Do you think there is a need to reduce the incidence of breed specific diseases?<br>f. Do you have any suggestions for what to do?
Knowledge and attitudes towards BOAS grading	1. Are you familiar with any specific measures to change	a. What initiatives are you familiar with?<br>b. How did you become familiar with these initiatives?

	the overall health of the breed?<br><br>2. Have you heard about the requirement for BOAS grading, and do you think it will have an effect?<br><br>3. Do you have suggestions on what can be done for that part of the breeding population which are outside the organized breeding associations and thus not subject to any requirements?	c. What do you think of these measures?<br><br>d. Are you familiar with the new BOAS graduation that the Danish Kennel Club has made mandatory from 1 August 2023?<br><br>e. What do you think of this requirement?<br><br>f. Do you think it will have an effect?<br><br>g. What do you think about the fact that only the French bulldogs bred within the Danish Kennel Club (approx. 12%) that are subject to the requirement for the BOAS graduation?
End		Is there anything else you would like to add?<br><br>Then we are slowly reaching the end of the interview. Finally, we would like to ask if we may contact you again when we have prepared our questionnaire.<br><br>Thank you very much for your participation – it has been a great help.
