# Supplementary material for: Perception of dog health and attitudes towards BOAS grading among Danish owners of French bulldog
Source: Front Vet Sci. 2025 Sep 15;12:1605505. doi: 10.3389/fvets.2025.1605505 (PMC12477691; doi:10.3389/fvets.2025.1605505)
Supplement: Supplementary file 2 [file Data_Sheet_2.pdf]

Translation of the questions used for the publication "Perception of health problems in French bulldogs and attitudes towards BOAS-grading among Danish owners".

The questions are part of a larger questionnaire, which also contained a section aimed specifically at breeders. These questions are omitted here.

The numbering of the questions here in the document is congruent with the questions in the data file

Introductory text:

The purpose of this survey is to explore the extent to which owners and breeders of French bulldogs believe that it is necessary to improve the breed's state of health. The study is conducted by veterinary students from the University of Copenhagen, and the results will be presented in our final MA thesis as well as in a scientific article.

If you want to read more about the background of the project, you can follow this link BOAS project

The survey takes about 10-15 minutes to complete. Your answers will be treated confidentially.

By marking "I accept and wish to participate", you agree that your answers may be used in the MA thesis as well as in the scientific article. To participate in the survey, you must be at least 18 years of age.

S_1

❍ I accept and wish to participate

❍ I do not wish to participate

- Thank you for your interest in the survey, but since you have stated that you do not wish to participate under the conditions stated, the survey will be closed.

❍ I am under the age of 18

- Thank you for your interest in the survey, but since you have stated that you are under the age of 18, you do not meet the requirements for participating.

S_3 Which gender do you identify as?

❍ Woman

❍ Man

❍ Other

❍ I do not want to answer

S_4 How old are you?

❍ 18-24 years old

❍ 25-34 years old

❍ 35-44 years old

❍ 45-54 years old

❍ 55-64 years old

❍ 65+ years old

S_5 Which region do you live in?

❍ Northern region of Jutland

❍ Middle region of Jutland

❍ Southern region of Denmark

❍ The region of Seeland

❍ The Capitol region

S_6 Which type of home do you live in?

❍ Terraced house

❍ Farm

❍ Detached house/villa

❍ Apartment

❍ Other (please describe) _____

S_8 What constitutes your household?

❍ I live alone

❍ I live with a partner/spouse

❍ I live with a partner/spouse and children

❍ I live with my children without a partner/spouse

❍ Other (please describe) _____

S_10 What is your highest completed level of education?

❍ Middle school

❍ High school

❍ Vocational school

❍ Short-cycle higher education (1-2 years)

❍ Medium-cycle higher education (2-4 years)

❍ Long-cycle higher education (more than 4 years)

❍ Other (please describe) _____

S_12 What is your current occupation?

❍ Self-employed

❍ Full-time wage earner (30 hours or morer)

❍ Part-time wage earner

❍ Unemployed

❍ On leave of absence

❍ Enrolled in education

❍ On early retirement, retiree, on disability pension

❍ On sick leave

❍ Other (please describe) _____

S_14 What is your household's total annual income before tax?

❍ < 100,000 DKK

❍ 100,000-199,999 DKK

❍ 200,000-299,999 DKK

❍ 300,000-399,999 DKK

❍ 400,000-499,999 DKK

❍ 500,000-599,999 DKK

❍ 600,000-699,999 DKK

❍ 700,000-799,000 DKK

❍ 800,000-899,999 DKK

❍ 900,000-1,000,000 DKK

❍ > 1,000,000 DKK

❍ I do not want to answer

❍ I do not know

S_17 Is it the first time you own a French bulldog?

❍ Yes

❍ No

If you own more than one French bulldog, please answer the following questions based on the dog that you acquired most recently.

S_18 Which sex is your dog?

❍ Intact female dog

❍ Neutered female dog

❍ Intact male dog

❍ Castrated male dog (surgically or medically)

S_19 How old is your dog?

❍ < 1 year old

❍ 1 year old

❍ 2 years old

❍ 3 years old

❍ 4 years old

❍ 5 years old

❍ 6 years old

❍ 7 years old

❍ 8 years old

❍ 9 years old

❍ 10 years old

❍ 11 years old

❍ 12 years old

❍ 13 years old

❍ 14 years old

❍ 15 years old

❍ 15+ years old

❍ I do not know

S_ 20 Does your dog have a pedigree?

❍ Yes (please state which one) _____

❍ No

❍ I do not know

S_21 How much did you pay for your dog?

❍ Nothing/it was given to me

❍ < 5,000 DKK

❍ 5,000-9,999 DKK

❍ 10,000-14,999 DKK

❍ 15,000-19,999 DKK

❍ 20,000-24,999 DKK

❍ 25,000-30,000 DKK

❍ > 30,000 DKK

❍ I do not want to answer

❍ I do not know

S_48 What is the likelihood that you will buy a French bulldog again?

❍ Very unlikely

❍ Unlikely

❍ Neither likely nor unlikely

❍ Likely

❍ Very likely

S_49 What is the likelihood that you will recommend a French bulldog to other potential buyers?

❍ Very unlikely

❍ Unlikely

❍ Neither likely nor unlikely

❍ Likely

❍ Very likely

To what extent do you consider the following diseases problematic in the breed?

	Not at all	To a lesser extent	To some extent	To a high extent	To a very high extent
S_100 Eye problems	❍	❍	❍	❍	❍
S_101 Spinal diseases	❍	❍	❍	❍	❍
S_102 Allergies	❍	❍	❍	❍	❍
S_103 Hip problems	❍	❍	❍	❍	❍
S_104 Breathing problems	❍	❍	❍	❍	❍
S_105 Reproductive problems	❍	❍	❍	❍	❍
S_106 Knee problems	❍	❍	❍	❍	❍
S_107 Skin fold infections	❍	❍	❍	❍	❍

Has your dog suffered from the following problems?

	Yes	No
S_82 Eye problems	❍	❍
S_83 Spinal diseases	❍	❍
S_84 Allergies	❍	❍
S_85 Hip problems	❍	❍
S_86 Breathing problems	❍	❍
S_87 Reproductive problems	❍	❍
S_88 Knee problems	❍	❍
S_89 Skin fold infections	❍	❍

Have you consulted a veterinarian about the following problems in your dog?

	Yes	No
S_91 Eye problems	❍	❍
S_92 Spinal diseases	❍	❍
S_93 Allergies	❍	❍
S_94 Hip problems	❍	❍
S_95 Breathing problems	❍	❍
S_96 Reproductive problems	❍	❍
S_97 Knee problems	❍	❍
S_98 Skin fold infections	❍	❍

S_120 On a scale from 1-5, where 1 = much less healthy, and 5 = much healthier, where would you place French bulldog compared to other dog breeds?

❍ 1 – Much less healthy

❍ 2 – Less healthy

❍ 3 – As average

❍ 4 - Healthier

❍ 5 – Much healthier

❍ I do not know

S_121 On a scale from 1-5, where 1 = much less healthy, and 5 = much healthier, where would you place your own dog compared to other French bulldogs?

❍ 1 – Much less healthy

❍ 2 – Less healthy

❍ 3 – As average

❍ 4 - Healthier

❍ 5 – Much healthier

❍ I do not know

Have you experienced any of the following in your dog?

	Yes	No
S_123 Snoring when asleep	❍	❍
S_124 Snoring when awake	❍	❍
S_125 Grunting	❍	❍
S_126 Wheezing	❍	❍

S_127 Regurgitation/vomiting	❍	❍
S_128 Sleep problems	❍	❍
S_129 Heat intolerance	❍	❍
S_130 Exercise intolerance	❍	❍

S_143 Are you familiar with the Danish Kennel Club's new requirement for BOAS grading of French bulldogs used for breeding?

❍ Yes, I am familiar with BOAS grading and I know what it entails

❍ Yes, I've heard of BOAS grading, but I'm not quite sure what it entails

❍ No, I've never heard of BOAS grading

S_52 A brief introduction opens for respondents who answered that they never heard of BOAS grading, or that they are not quite sure what it entails:

• BOAS grading is a method used to assess the degree of BOAS (brachycephalic syndrome) which is a disease-complex characterized by breathing problems. At present, the method can be used for Bulldogs, French bulldogs, and Pugs.

• At a BOAS grading, a certified veterinarian makes an assessment of the opening of the nostrils and listens to the dog's breathing before and after a 3-minute running test.

• The dog is assigned a grade between 0 and 3, with 0 is given to a clinically unaffected dog with no signs of breathing problems, and 3 is given to a clinically affected dog with severe signs of breathing problems.

• Only dogs with grade 0 and 1 are recommended for breeding, while dogs with grade 2 can be used if the breeding partner has grade 0 or 1. In this way, a reduction in the occurrence of BOAS is expected.

• From 1 August 2023, the Danish Kennel Club has made it mandatory that all French bulldogs used for breeding must have completed a BOAS grading with approved results in order to be able to register their puppies. However, there is currently no requirement for BOAS grading of French bulldogs outside the Danish Kennel Club.

Where did you hear about the requirement for BOAS grading? (multiple answer options)

S_144_1 Danish Kennel Club/Breed club

S_144_2 Social media

S_144_3 News media

S_144_4 Friends/relatives

S_144_5 The veterinarian

S_144_6 Breeders

S_144_7 I do not know

S_144_8 Other (please describe) _____

How do you agree with the following statements?

	Completely disagree	Partly disagree	Neither agree nor disagree	Partly agree	Completely agree	Do not know
S_145 BOAS grading is a good initiative	❍	❍	❍	❍	❍	❍
S_146 BOAS grading promotes the health of the breed in the long term	❍	❍	❍	❍	❍	❍
S_147 The requirement for BOAS grading should be introduced across all pedigree organizations	❍	❍	❍	❍	❍	❍
S_148 The BOAS grading requirement should apply to all French bulldogs used for breeding	❍	❍	❍	❍	❍	❍

S_149 The requirement for BOAS modulation should apply to all French bulldogs, whether they are used for breeding or not	❍	❍	❍	❍	❍	❍
S_150 The requirement for BOAS modulation should be regulated by legislation	❍	❍	❍	❍	❍	❍

S_151 Is your dog(s) BOAS graded?

❍ Yes

❍ No

❍ I do not know

S_152 How likely is it that you will have your current dog(s) BOAS graded?

❍ Very unlikely

❍ Unlikely

❍ Neither likely nor unlikely

❍ Likely

❍ Very likely

❍ I do not know

S_154 How likely is it that you in the future will buy a puppy whose parents have been BOAS graded?

❍ Very unlikely

❍ Unlikely

❍ Neither likely nor unlikely

❍ Likely

- ❍ Very likely
- ❍ I do not know
