# Supplementary material for: Perception of dog health and attitudes towards BOAS grading among Danish owners of French bulldog
Source: Front Vet Sci. 2025 Sep 15;12:1605505. doi: 10.3389/fvets.2025.1605505 (PMC12477691; doi:10.3389/fvets.2025.1605505)
Supplement: Supplementary file 3 [file Data_Sheet_3.pdf]

Supplementary Material 3:

Ordinal logistic regression analyses of socio-demographic aspects and animal-related aspects on owners' estimation of the health status of French bulldog compared to other breeds

(χ^2 (5) = 39.633, P<0.001)					
	**B**	**Std. Error**	**Wald Chi-Square**	**df**	**Sig.**
Gender [ref.cat.: Man]	-,485	,3324	2,131	1	,144
Age	,034	,0774	,196	1	,658
Total score of perceived health problems	-,424	,0730	33,694	1	**<.001**
Frist time having a French Bulldog [ref.cat.: No]	,096	,1979	,123	1	,725
Cost of the dog	-,067	,0638	1,109	1	,292

*Bonferroni correction was applied for significant results; as there are four tests being made, alpha was divided by 4 (N = 4): 0.05/4 = 0.0125, i.e., each test is tested against a level of 0.0125. Significant p-values are highlighted in bold.

Ordinal logistic regression analyses of socio-demographic aspects and animal-related aspects on owners' estimation of the health status of their own dog compared to other French bulldogs

(χ^2 (5) =137,846, P<0.001)					
	**B**	**Std. Error**	**Wald Chi-Square**	**df**	**Sig.**
Gender [ref.cat.: Man]	,156	,3046	,261	1	,609
Age	-,031	,0738	,174	1	,677
Total score of perceived health problems	-,810	,0772	110,184	1	**<,001**
Frist time having a French Bulldog [ref.cat.: No]	,220	,1922	1,308	1	,253
Cost of the dog	,081	,0629	1,655	1	,198

*Bonferroni correction was applied for significant results; as there are four tests being made, alpha was divided by 4 (N = 4): 0.05/4 = 0.0125, i.e., each test is tested against a level of 0.0125. Significant p-values are highlighted in bold.
