# Supplementary material for: Perception of dog health and attitudes towards BOAS grading among Danish owners of French bulldog
Source: Front Vet Sci. 2025 Sep 15;12:1605505. doi: 10.3389/fvets.2025.1605505 (PMC12477691; doi:10.3389/fvets.2025.1605505)
Supplement: Supplementary file 4 [file Data_Sheet_4.pdf]

**Supplementary Material 4:**
Ordinal logistic regression analyses of owner-related aspects, perceived health status of French bulldogs of their own dogs, as well as their attitudes towards BOAS grading on the likelihood of having the current dog BOAS-graded in the future

| ($\chi^2$ (7) = 56,338, P<0.001) | | | | | |
|---|---|---|---|---|---|
| | **B** | **Std. Error** | **Wald Chi-Square** | **df** | **Sig.** |
| Gender [ref.cat.: Man] | -,293 | ,3757 | ,607 | 1 | ,436 |
| Age | -,486 | ,0915 | 28,229 | 1 | **<,001** |
| Total score of perceived BOAS-related health problems | -,053 | ,0637 | ,686 | 1 | ,408 |
| Owners' estimation of the health status of French bulldog compared to other breeds | ,034 | ,1568 | ,047 | 1 | ,828 |
| Owners' estimation of the health status of their own dog compared to other French bulldogs | ,207 | ,1152 | 3,241 | 1 | ,072 |
| BOAS grading is a good initiative | ,382 | ,1565 | 5,957 | 1 | ,015 |
| BOAS grading should be mandatory for all French bulldogs used for breeding, regardless of pedigree | ,312 | ,0931 | 11,204 | 1 | **<,001** |

*Bonferroni correction was applied for significant results; as there are six tests being made, alpha was divided by 6 (N = 6): 0.05/6 = 0.008, i.e., each test is tested against a level of 0.008. Significant p-values are highlighted in bold.

Ordinal logistic regression analyses of owner-related aspects, perceived health status of French bulldogs of their own dogs, as well as their attitudes towards BOAS grading on the likelihood of preferring a puppy from BOAS-graded parents when reacquiring a French bulldog

| ($\chi^2$ (7) = 64,290, P<0.001) | | | | | |
|---|---|---|---|---|---|
| | **B** | **Std. Error** | **Wald Chi-Square** | **df** | **Sig.** |
| Gender [ref.cat.: Man] | ,006 | ,3698 | ,000 | 1 | ,986 |
| Age | -,012 | ,0969 | ,015 | 1 | ,902 |
| Total score of perceived BOAS-related health problems | ,010 | ,0726 | ,020 | 1 | ,888 |
| Owners' estimation of the health status of French bulldog compared to other breeds | -,049 | ,1687 | ,083 | 1 | ,773 |
| Owners' estimation of the health status of their own dog compared to other French bulldogs | ,153 | ,1337 | 1,303 | 1 | ,254 |
| BOAS grading is a good initiative | ,771 | ,1622 | 22,595 | 1 | **<,001** |
| BOAS grading should be mandatory for all French bulldogs used for breeding, regardless of pedigree | ,387 | ,0957 | 16,386 | 1 | **<,001** |

*Bonferroni correction was applied for significant results; as there are six tests being made, alpha was divided by 6 (N = 6): 0.05/6 = 0.008, i.e., each test is tested against a level of 0.008. Significant p-values are highlighted in bold.
