# Supplementary material for: Perception of dog health and attitudes towards BOAS grading among Danish owners of French bulldog
Source: Front Vet Sci. 2025 Sep 15;12:1605505. doi: 10.3389/fvets.2025.1605505 (PMC12477691; doi:10.3389/fvets.2025.1605505)
Supplement: Supplementary file 5 [file Data_Sheet_5.pdf]

Supplementary Material 5:

Ordinal logistic regression analyses of owner-related aspects, perceived health status of French bulldog, of their own dog, as well as their attitudes towards BOAS grading on the likelihood of reacquiring a French bulldog

($\chi^2(7)=66{,}536$, P<0.001)					
	**B**	**Std. Error**	**Wald Chi-Square**	**df**	**Sig.**
Gender [ref.cat.: Man]	-,272	,4516	,363	1	,547
Age	,222	,0990	5,027	1	,025
Owners' estimation of the health status of French bulldog compared to other breeds	,931	,1907	23,808	1	**<,001**
Owners' estimation of the health status of their own dog compared to other French bulldogs	,255	,1416	3,239	1	,072
BOAS grading is a good initiative	-,160	,1811	,784	1	,376
BOAS grading should be mandatory for all French bulldogs used for breeding, regardless of pedigree	-,049	,1024	,226	1	,635
Total score of perceived BOAS-related health problems	-,244	,1029	5,617	1	,018

*Bonferroni correction was applied for significant results; as there are six tests being made, alpha was divided by 6 (n = 6): 0.05/6 = 0.008, i.e., each test is tested against a level of 0.008. Significant p-values are highlighted in bold.

Ordinal logistic regression analyses of owner-related aspects, perceived health status of French bulldogs of their own dogs, as well as their attitudes towards BOAS grading on the likelihood of recommending a French bulldog to others

(χ^2(7)=103,670, P<0.001)					
	**B**	**Std. Error**	**Wald Chi-Square**	**df**	**Sig.**
Gender [ref.cat.: Man]	-,246	,3416	,517	1	,472
Age	,054	,0792	,469	1	,493
Owners' estimation of the health status of French bulldogs compared to other breeds	1,016	,1584	41,149	1	**<,001**
Owners' estimation of the health status of their own dog compared to other French bulldogs	,250	,1174	4,543	1	,033
BOAS grading is a good initiative	-,034	,1327	,064	1	,800
BOAS grading should be mandatory for all French bulldogs used for breeding, regardless of pedigree	-,118	,0847	1,925	1	,165
Total score of perceived BOAS-related health problems	-,242	,0885	7,494	1	**,006**

*Bonferroni correction was applied for significant results; as there are six tests being made, alpha was divided by 6 (n = 6): 0.05/6 = 0.008, i.e., each test is tested against a level of 0.008. Significant p-values are highlighted in bold.
